# Pathophysiological, Cellular, and Molecular Events of the Vascular System in Anaphylaxis

**DOI:** 10.3389/fimmu.2022.836222

**Published:** 2022-03-08

**Authors:** Emilio Nuñez-Borque, Sergio Fernandez-Bravo, Alma Yuste-Montalvo, Vanesa Esteban

**Affiliations:** ^1^ Department of Allergy and Immunology, Instituto en Investigación Sanitaria - Fundación Jiménez Díaz (IIS-FJD), Universidad Autónoma de Madrid (UAM), Madrid, Spain; ^2^ Faculty of Medicine and Biomedicine, Alfonso X El Sabio University, Madrid, Spain

**Keywords:** anaphylaxis, immune system, vascular system, endothelium, vasodilation, vascular permeability

## Abstract

Anaphylaxis is a systemic hypersensitivity reaction that can be life threatening. Mechanistically, it results from the immune activation and release of a variety of mediators that give rise to the signs and symptoms of this pathological event. For years, most of the research in anaphylaxis has focused on the contribution of the immune component. However, approaches that shed light on the participation of other cellular and molecular agents are necessary. Among them, the vascular niche receives the various signals (e.g., histamine) that elicit the range of anaphylactic events. Cardiovascular manifestations such as increased vascular permeability, vasodilation, hypotension, vasoconstriction, and cardiac alterations are crucial in the pathophysiology of anaphylaxis and are highly involved to the development of the most severe cases. Specifically, the endothelium, vascular smooth muscle cells, and their molecular signaling outcomes play an essential role downstream of the immune reaction. Therefore, in this review, we synthesized the vascular changes observed during anaphylaxis as well as its cellular and molecular components. As the risk of anaphylaxis exists both in clinical procedures and in routine life, increasing our knowledge of the vascular physiology and their molecular mechanism will enable us to improve the clinical management and how to treat or prevent anaphylaxis.

**Key Message:**

Anaphylaxis, the most severe allergic reaction, involves a variety of immune and non-immune molecular signals that give rise to its pathophysiological manifestations. Importantly, the vascular system is engaged in processes relevant to anaphylactic events such as increased vascular permeability, vasodilation, hypotension, vasoconstriction, and decreased cardiac output. The novelty of this review focuses on the fact that new studies will greatly improve the understanding of anaphylaxis when viewed from a vascular molecular angle and specifically from the endothelium. This knowledge will improve therapeutic options to treat or prevent anaphylaxis.

## Introduction

Anaphylaxis is a systemic reaction with a range of clinical manifestations due to the varying involvement of several organs and systems. At the cellular and molecular levels, most existing research focuses exclusively on the immune component. However, interactions between vascular and immunological microenvironments are responsible for most manifestations of anaphylaxis, including the most severe. Therefore, precise understanding of the cellular communication between immune and resident cells within the complex pathophysiology of this pathological event is necessary. This review provides an overview of the human vascular system in anaphylaxis and seeks further understanding of the dysregulation of its underlying processes.

## Anaphylaxis

Anaphylaxis is considered the most serious manifestation of allergic disorders. The World Health Organization defines it as “a severe, life-threatening systemic hypersensitivity reaction characterized by being rapid in onset with potentially life-threatening airway, breathing, or circulatory problems and is usually, although not always, associated with skin and mucosal changes” (1). In the United States of America (USA) alone, annual expenditures due to anaphylaxis total 1.8 billion dollars between direct and indirect costs (2). The World Allergy Organization has found that its incidence and prevalence have increased over the last decade. In 2020, global incidence was 50–112 episodes per 100,000 people, and its lifetime prevalence was between 0.3% and 5.1%. Moreover, although the mortality rate remains low (0.05–0.51, 0.03–0.32, and 0.09–0.13 per million people/year for drug-, food-, and venom-induced lethal reactions, respectively), the rate of recurrence was 26.5%–54.0% over a follow-up time of 1.5–25 years (1, 3). However, current data underestimate the true actual rate due to factors such as the lack of a common definition of anaphylaxis or discrepancies in the methodologies used in epidemiological studies (1, 4). In addition, diagnosis of this pathologic event is based on clinical symptoms that are shared with other diseases, causing anaphylactic reactions to be underdiagnosed (5). Thus, in order to complement diagnosis, molecular markers are also evaluated. Currently, the main biomarker used in clinical practice is serum tryptase, a molecule released by the effector cells of anaphylaxis. The diagnostic reference threshold for this measurement has undergone considerable modifications to the current value of 11.4 μg/l. However, its clinical utility has several drawbacks since serum tryptase is also elevated in other conditions such as mast cell disorders (6). In addition, it is not increased in most patients who develop an anaphylactic reaction (7). This could be due to the sample collection as the peak of this protein is obtained 2 h after the reaction (8). Moreover, most studies have now demonstrated the importance of considering the basal value of serum tryptase at least 24 h after the episode (9, 10). Specifically, the increase of 20% plus 2 μg/l in the acute condition with respect to baseline revealed a substantial improvement in the diagnosis of anaphylaxis (11). Therefore, correct sampling and personalized diagnosis of each individual, as well as the need to find new biomarkers, are crucial for the correct management of this life-threatening reaction.

Several triggers can induce anaphylaxis, the most common being foods, drugs, and Hymenoptera venoms, although in some cases the cause of the reaction is unknown (idiopathic) (12–14). In particular, the etiological distribution of anaphylaxis differs with age since the most common triggers are drugs and insect stings in adults, while food is the main culprit in children (5, 15). However, regardless of the allergen, the signs and symptoms of anaphylaxis are the same and affect several systems. Cutaneous symptoms (e.g., localized urticaria, erythema, angioedema, pruritus) are the most common manifestations and are present in 80% of cases. Other systems may be affected, such as the gastrointestinal (e.g., emesis, diarrhea), nervous (e.g., confusion, drowsiness, seizure), respiratory (e.g., dyspnea, cough, wheezing, bronchoconstriction), and circulatory (e.g., palpitations, tachycardia, hypotension) (16). The cardiovascular system is highly involved during mild anaphylactic reactions and plays a key role in the most severe cases, in which anaphylactic shock could take place (17, 18). Deterioration of the circulatory system due to age, other diseases, and treatments administered such as angiotensin-converting enzyme inhibitors are some of the main risk factors associated with fatal reactions (19–21). Therefore, the cardiovascular system is essential in the development of the reaction and a key target to developing future therapeutic strategies.

## Immune System in Anaphylaxis

### Cellular and Molecular Mechanisms Mediated by IgE

**Figure 1:**
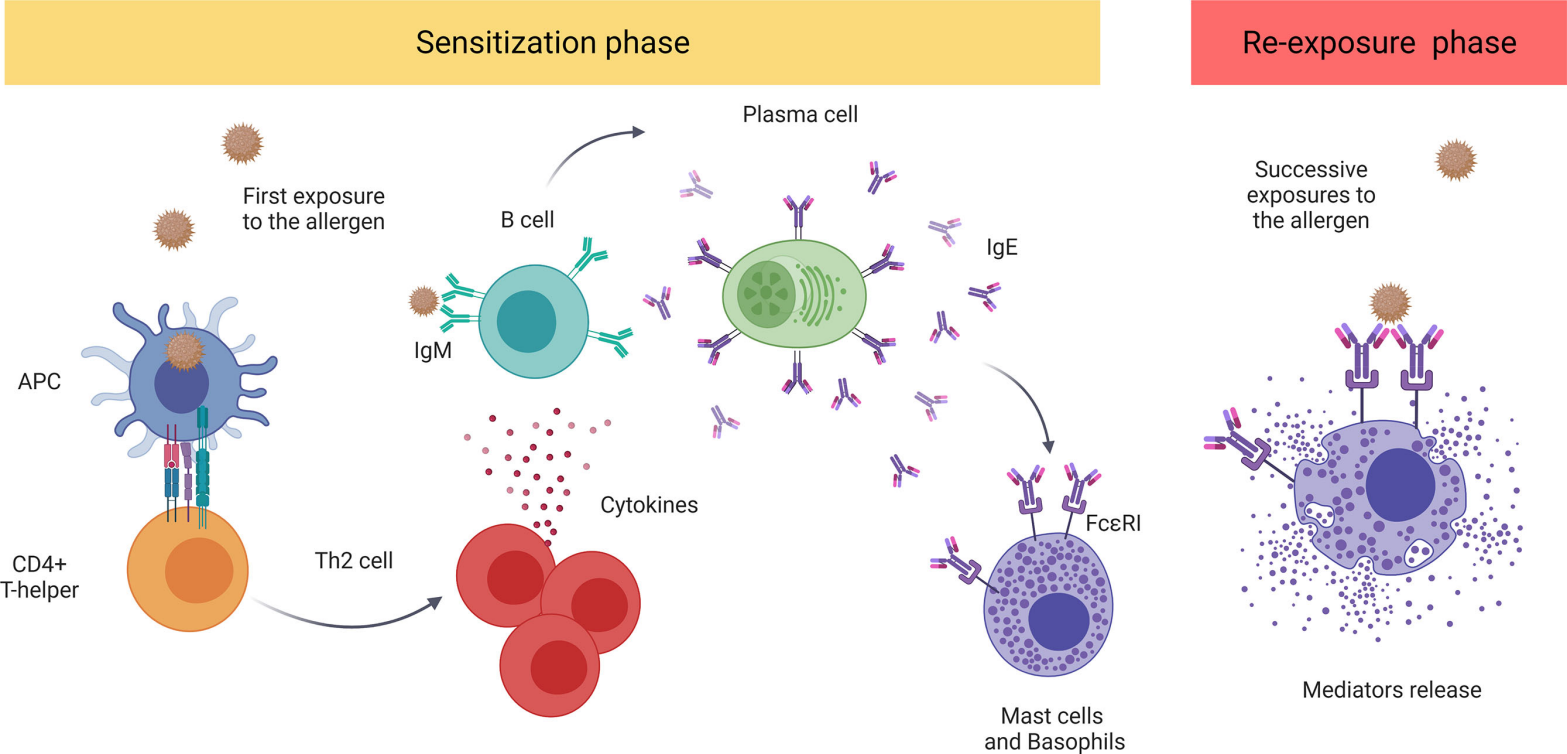
The classic IgE-mediated immune mechanism. The sensitization process initiates with first exposure to an allergen. The antigen-presenting cells (APCs) capture and present the processed antigen to CD4+T cells inducing their polarization to a Th2 phenotype. These stimulate B cells which, afterward, produce and release antigen-specific IgE antibodies that bind to FcϵRI. Future re-exposure(s) to the allergen induces the cross-linking of FcϵRI-bound allergen–IgE complexes, activating and inducing the degranulation of mediators by mast cells and basophiles. These include histamine, platelet-activating factor (PAF), and tryptase, among others. APC, antigen-presenting cell; PAF, platelet-activating factor.

The major molecular mechanism underlying anaphylaxis is the classic allergic IgE-mediated reaction involving mast cells and basophils (22). Mast cells reside in all vascularized tissues, and studies have shown a correlation among the severity of the reaction, early degranulation of mast cells, and release of mediators (23–25). On the other hand, basophils are also granular immune cells, although they are blood-circulating leukocytes and not tissue-resident cells, unlike mast cells (26, 27). Recent studies suggest that basophils play a key role in food-mediated anaphylaxis. However, since the activation of these cells is complementary to that of mast cells, their contribution to human anaphylaxis remains a pivotal point of study (28, 29). Both cell types present the high-affinity IgE receptor (FcϵRI) on their surface and are considered the main effectors in this pathologic event (22). Mechanistically, when the individual is first exposed to an allergen, the immune sensitization process is initiated, triggering the production of antigen-specific IgE antibodies. In successive reexposures to the antigen, there is cross-linking of FcϵRI-bound allergen-IgE complexes, activating effector cells and giving rise to the release of a large number of mediators (12, 13, 30) (**Figure 1**).

### IgE-Independent Cellular and Molecular Mechanisms

Interestingly, after experiencing an anaphylactic reaction, some patients have no detectable levels of allergen-specific IgE (9, 31, 32). This happening means that a considerable percentage of subjects do not show evidence of IgE-dependent immune activation, so other cells and processes must be involved (33–35). Due to the similarity between mouse and human immune systems, the use of murine models has been essential in elucidating the role of other mechanisms in anaphylaxis. Among them, the IgG pathway appeared as the main alternative immune process described in anaphylactic reactions (5, 35, 36). Unlike the IgE mechanism, the mechanism guide by IgGs seems to require higher levels of specific IgG and antigens, presumably due to the lower affinity of FcγR compared with FcϵRI (33). Nowadays, it is well established that IgG antibodies can induce anaphylaxis by binding to their different receptors (FcγR) (37). These ones are found in mast cells, basophils, neutrophils, monocytes, and macrophages conforming to the major cellular types activated by this alternative pathway. The cellular consequence is elicited reactions due to the release of abundant mediators (33, 38–41). One of the common mediators released, in response to both IgE and IgG molecules, is the platelet-activating factor (PAF) which is released from all the subsets coming from a myeloid progenitor (**Table 1**).

**Table 1:** Anaphylaxis-associated products and their main cellular sources are listed.

	Molecules	Source	References
Vasoactive agents	Histamine	MC, BAS, NEUT	(42, 43)
	Bradykinin	P	(44, 45)
	NO	EC	(46, 47)
	Endothelin-1	EC, SMC, hMC, MAC, MON	(47–50)
Proteases	Tryptase	MC, BAS	(46, 51)
	Carboxypeptidase A	MC, BAS	(21, 51, 52)
	Chymase	MC	(9, 42, 46)
	Plasminogen activator	MC	(9)
	Cathepsin G	MC, NEUT, EOS, BAS	(9, 26, 46, 53, 54)
	Elastase	NEUT	(55)
Lipidic molecules	LTB4, LTC4, LTD4, LTE4	MC, BAS, NEUT	(9, 26, 42, 46, 51, 56)
	PGD2	MC, NEUT, MON, MAC, PLAT	(51, 56–58)
	PGE2	MC, BAS MAC, EOS, PLAT, SMC	(26, 58, 59)
	PGF2	MC, PLAT	(26, 58)
	TXA2	MC, EOS, PLAT,	(26, 42, 58)
	TXB2	MC	(60, 61)
	Prostacyclin	MC, ECs, SMC, PLAT	(48, 58, 62, 63)
	PAF	MC, BAS, NEUT, EOS, MON, MAC, PLAT, EC	(43, 52, 64–67)
	S1P	MC, PLAT, ERY, EC	(68–70)
Derived from	FXII, PK, HK Thrombin	P	(44)
		P	(44)
Contact, Coagulation and Complement systems activation	C3a	P	(44)
	C5a	P	(44)
	Heparine	MC, BAS	(9)
	Chondroitin sulfate	MC, BAS	(9)
	Renin	hMC	(9)
Cytokines and chemokines	TNF α	MC, MAC, NEUT, BAS	(26, 42, 56, 71, 72)
	TGF-β	MC, EOS	(22, 26, 46)
	IFN-γ	NEUT, LT, NK	(56, 73, 74)
	G-CSF	MC, MAC, MONO, NEUT, EC	(42, 75)
	M-CSF	MC, MONO, EC	(42, 76)
	GM-CSF	MC, BAS, EC	(26, 42, 77)
	CCL-2	MC, EC, NEUT, MAC, MON	(22, 46, 78–82)
	CCL-5	MC, EOS, MON	(26, 46, 83)
	Stem cell factor	MC	(9, 46)
	TWEAK	EC	(84)
	IgE-dependent histamine releasing factor	BAS	(9)
	MIP-1α	BAS	(9)
	Platelet factor 4 (PF4)	PLAT	(64, 85)
Interleukins	IL-1β	MC, NEUT	(42, 56)
	IL-3	MC, EOS	(26, 42)
	IL-4	MC, BAS, EOS	(9, 22, 42, 46)
	IL-5	MC, EOS	(9, 22, 26, 46)
	IL-6	MC, MAC, NEUT, BAS	(9, 21, 42, 46, 72, 86)
	IL-8	MC, NEUT, EOS	(9, 22, 26, 42, 56)
	IL-10	MC, EOS	(22, 26, 42, 46)
	IL-13	MC, BAS, EOS	(21, 26, 42, 46)
	IL-16	MC, EOS	(26, 42)
	IL-18	MC, EOS	(26, 42)
	IL-22	MC	(42)
	IL-33	MC, BAS	(21)
Proteins	VEGF	MC, MAC, PLAT	(46, 71, 87)
	Basogranulin	BAS	(9, 52)
	L-Selectin	NEUT	(86)
	MPO	NEUT, MON	(56, 86, 88)
	MBP	EOS	(26)
	β-TG	PLAT	(64)

NO, nitric oxide; LTB4, leukotriene B4; LTC4, leukotriene C4; LTD4, leukotriene D4; LTE4, leukotriene E4; PGD2, prostaglandin D2; PGE2, prostaglandin E2; PGF2, prostaglandin F2; TXA2, thromboxane A2; TXB2, thromboxane B2; PAF, platelet activating factor; S1P, sphingosine 1 phosphate; FXII, coagulation factor XII; PK, plasma kallikrein; HK, kininogen; TNF, tumor necrosis factor; TGF, transforming growth factor; IFN, interferon; G-CSF, granulocyte colony-stimulating factor; M-CSF, macrophage colony-stimulating factor; GM-CSF, granulocyte-macrophage colony-stimulating factor; TWEAK, TNF-related weak inducer of apoptosis; MIP, macrophage inflammatory proteins; PF4, platelet factor 4; IL, interleukin; VEGF, vascular endothelial growth factor; MPO, myeloperoxidase; MBP, major basic protein; β-TG, beta-thromboglobulin; MC, mast cells; hMC, heart mast cells; EOS, eosinophils; BAS, basophils; MAC, macrophages; MON, monocytes; EC, endothelial cells; PLAT, platelets; NEUT, neutrophils; ERY, erythrocytes; SMC, smooth muscle cells; NK, natural killer cells; LT, T lymphocytes; P, plasma.

Specifically, the implication of both monocytes and macrophages has been demonstrated in passive and active systemic anaphylaxis (12, 89, 90). However, the role of neutrophils has gained much relevance as key cellular players eliciting anaphylactic reactions (43). Their contribution was firstly observed in experimental mouse models which suggested that a similar pathway could be operative in human reactions (43, 56). In fact, evidence in patients has shown elevated circulating serum levels of neutrophil elastase and myeloperoxidase (the major mediators stored in their granules). These results supported the existence of a neutrophil-associated IgG molecular mechanism associated with drug-induced anaphylaxis (86, 91). Therefore, neutrophils and IgG arise as important factors in the etiopathogenesis of the reaction but also lead to the possibility of constituting new biomarkers of anaphylaxis.

Other reactions triggered by drugs (e.g., opioids, vancomycin) are also capable of activating mast cells and basophils as well as degranulation led by the Mas-related G-protein coupled receptor member X2 (MRGPRX2) (92–95). This relevant pathway would explain those reactions absent of immunoglobulins, and therefore huge effort is being realized to elucidate its relevance in the setting of anaphylaxis (35, 96, 97). Even further, external factors (e.g., physical exercise, exposure to cold, or ultraviolet radiation) contribute as cofactors in the reactions (12, 34, 41, 98).

Concerning the participation of other immune cells in these events, accumulation of eosinophils was detected in passive cutaneous anaphylactic reactions in guinea pigs, as well as in spleens and coronary arteries from anaphylactic human cadavers (99, 100). Specifically, these cells express on their surface both receptor types: FcγR and FcϵR (26). Moreover, the contribution of platelets to the reaction by the release of important mediators has been also evidenced through IgE- and IgG-dependent mechanisms (64, 101). Therefore, it seems that a big portion of myeloid cells activated in anaphylaxis (endotypes) exists, which are associated with different anaphylactic phenotypes and biomarkers (5).

## Soluble Mediators and Cascades in Anaphylaxis

Overall, a large number of anaphylactic mediators coming from different cellular sources are released both into the bloodstream and in local microenvironments (**Table 1**). The nature of these molecules is very diverse: vasoactive agents, proteases, lipidic particles, cytokines, chemokines, interleukins, hormones, and neurotransmitters, among others. In addition, due to the homeostatic imbalance and acute inflammatory state characteristic of anaphylaxis, complement and contact/coagulation cascades are involved in the pathophysiology of this event leading to the release of numerous intermediate products (42, 44, 102).

Classically, the best-characterized mediators have been classified into preformed and newly synthesized. Those stored in cytoplasmic granules contain highly sulfated polysaccharides (heparin or other proteoglycans), tryptase, chymase, histamine, and PAF, being mainly released from mast cells and basophils (22, 24). However, other cellular providers, such as neutrophils or platelets, also supply key molecular effectors as PAF to the reaction (103). Molecules derived from arachidonic acid, interleukins, chemokines, and/or cytokines are also massively released (42, 104, 105). In summary, the diversity of triggers and/or signals described upstream to the cellular activation induces different degranulation strategies in mast cells (106). Probably, it also occurs with other participating cells of the anaphylactic reaction. Into the downstream signaling following cellular activation, the influx of mediators causes the recruitment of other immune cells but also the activation of resident cells that contribute, in turn, with a multitude of other anaphylactic products amplifying the molecular and cellular signaling (107, 108).

In relation, multitudes of soluble products from both the contact (bradykinin) and the complement system (C3a, C4a, C5a) are also released in the pool of mediators (109, 110). The activation of the coagulation, fibrinolytic, contact, and complement system pathways is highly involved in anaphylaxis. Peptides C3a, C4a, and C5a derived from the proteolysis of the C3, C4 and C5 components of the complement system are considered, along IgG molecules, the main elicitors of non-IgE anaphylactic reactions. These molecules, often known as anaphylatoxins, induce mast cell degranulation through its specific receptors on their surface (35). On the other hand, contact and coagulation/fibrinolytic systems are closely related to each other and are involved in the so-called non-immunologic anaphylactic reactions. Coagulation factors such as kininogen, fibrinogen, and factors V and VII are decreased in anaphylactic patients (44).

In addition, other molecular players such as extracellular vesicles (EVs), microRNAs, and metabolites are just starting to gain relevance as surrogate biomarkers in anaphylaxis but also participating in their underlying molecular pathways (13, 111–113).

Altogether, this large variety of molecular signaling pathways contributes to amplify the number and heterogeneity of processes occurring in these pathological events.

## The Cardiovascular System

The cellular components of the vascular compartment are both target and effector resident cells in anaphylaxis. Thus, the following section expands on the morphological and functional particularities of the cardiovascular system in order to broaden its knowledge in anaphylactic reactions. The circulatory system is composed of a set of interconnected tubular organs that form a closed circuit in the human body. It is divided into the blood vascular system, which carries blood, and the lymphoid vascular system, which transports lymph (114). However, in this review, we will only focus on the former.

**Figure 2:**
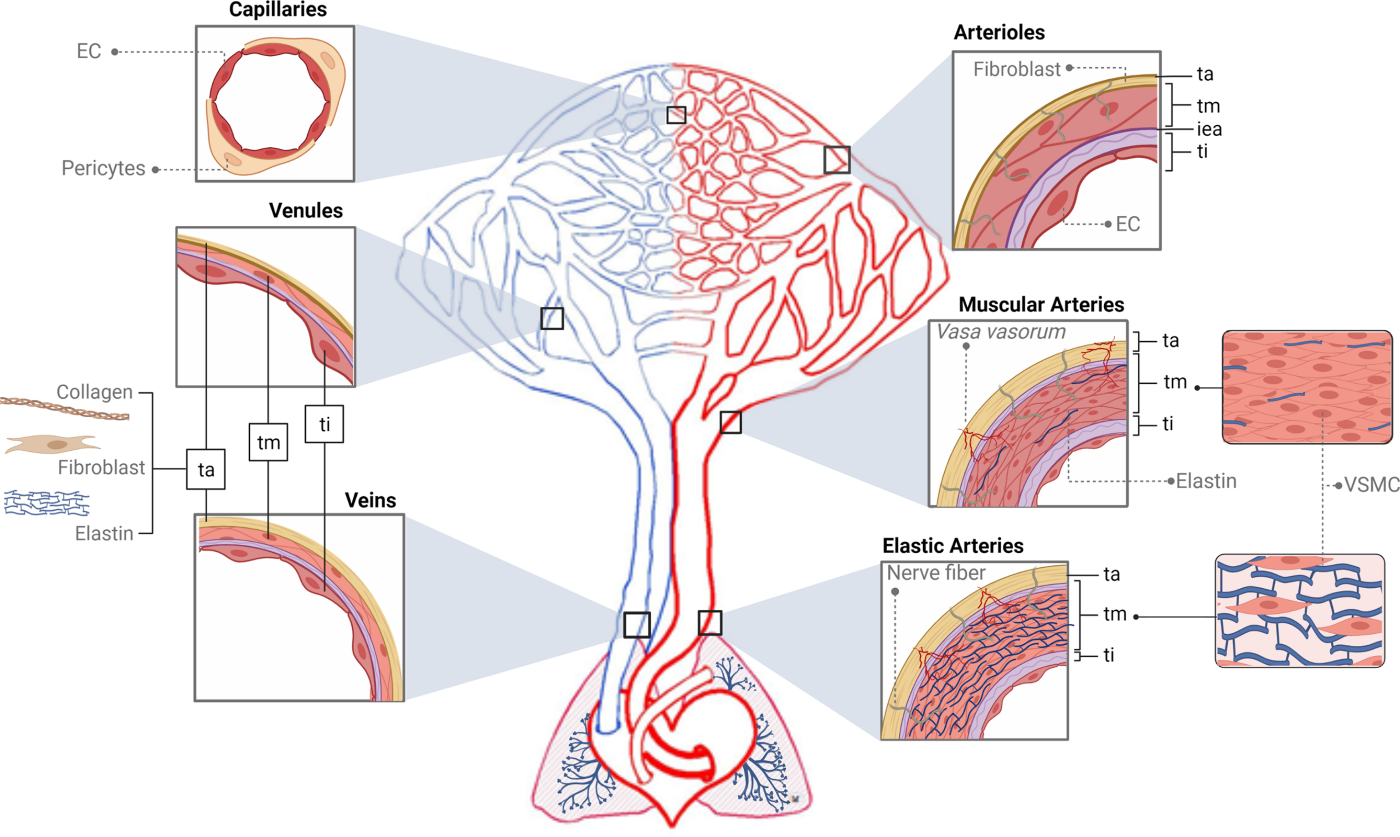
Structure of the vascular tree. The *tunica intima* (*ti*) is the inner area of the vessels, which interacts with the content of the lumen and is supported by a basement membrane surrounded by connective tissue and a fibroelastic layer called the internal elastic lamina (iea, purple color). The *tunica media* (*tm*) is composed of several layers of vascular smooth muscle cells (VSMCs) and elastin fibers supported mainly by a collagen matrix. The *tunica adventitia* (*ta*) is formed by collagen, fibroblasts, and elastin. In red, first big vessels arising from the heart are the elastic arteries (EA). The *tm* of EA is thick and made up of several elastic sheets interconnected by elastin cords and smooth muscle fibers. The *ta* is relatively thin and presents both nerve fibers and *vasa vasorum*. In the EA branch, the elastic component begins to be replaced by the muscular one giving rise to the muscular arteries (MA) that present abundant VSMCs. Structurally, their *tm* is formed of several layers of VSMCs, including collagen, elastic, and reticular fibers. The continuous bifurcation of MA leads to narrower vessels called arterioles presenting a robust *tm* made up of only a few layers of VSMCs. Particularly, their iea is thin and fenestrated and the *ta* does not present *vasa vasorum*. Next, capillaries (Cap), due to their thinness, do not present tunics but can be surrounded by pericytes. In blue, blood returns to the heart through the venous system. Venules (V) are similar to the Cap and are made up of endothelial cells, a basal lamina, collagen fibers, and pericytes. When these V move away from the Cap, they form the muscular V, presenting an appreciable *tm* composed mainly of VSMCs and, occasionally, a *ta*. Next, the V give rise to veins whose structure presents the three layers but thinner than arteries’. The *ti* is similar to the V, a *tm* composed of a few fibers of VSMCs and a complete *ta*. *Ti, tunica intima*; tm, *tunica media*; *ta, tunica adventitia*; iea, internal elastic lamina; EA, elastic arteries; MA, muscular arteries; VSMCs, vascular smooth muscle cells; Cap, capillaries; V, venules.

The functions of the blood vascular system are as follows: transportation of substances entering the body from the external environment (mainly nutrients and oxygen), transfer of molecules such as hormones, hydraulic force generation, regulation of heat, ultrafiltration in the kidney, and defense through the transport of immune cells and mediators (115). This circuit is made up of the heart and a number of different types of vessels that distribute blood to the organs. Elastic arteries emerge from the heart and branch out to the muscular arteries and arterioles. The latter give rise to the capillaries, and from these, venules and veins return blood to the heart (114, 116, 117) (**Figure 2** and **Box 1**).

Box 1 **Understanding the structure and function of the main vascular regions.**

***The heart*** is an organ located in the central part of the thoracic cavity and made up of two thin-walled atria and two thick-walled ventricles. The right and left parts of the heart are separated by a septum, so blood from one side does not mix with the other (118). However, the atria and ventricles in both areas contract in a coordinated manner. The function of this organ is to pump blood so that it may be distributed throughout the vessels in the rest of the body. For this to happen, a sequence of muscular contractions (systole) and relaxations (diastole) is carried out due to changes in the levels of intracellular calcium in cardiac myocytes, giving rise to the cardiac cycle (119–121). Increasing evidence indicates that the human heart is a target of cardiac anaphylaxis, and in which human heart mast cells (HHMC) play a key role (122).

***Elastic arteries*** (e.g., aorta, pulmonary artery, carotids) arise from the heart and transport blood to the muscular arteries. In each ventricular systole, the heart pumps a very large volume of blood, causing distension of the elastic walls, which accumulate the potential energy released during the diastole. Therefore, they push the blood when the heart is relaxed, acting as a secondary propulsion pump complementing the heart and resulting in a continuous flow (114, 117).

***Muscular arteries*** (e.g., brachial artery, femoral artery) are the most abundant arteries in the human body. They usually appear near the organs, and their function is to distribute the blood through the different regions of the body since, by contracting their muscular component, they can regulate the size of their lumen (114, 116, 117, 123).

***Arterioles*** are the vessels responsible for regulating blood flow since their muscle fibers modulate the lumen of the vessel (114, 116, 117, 124). Specifically, arterioles have a precapillary sphincter with a conical shape that also allows blood flow regulation (125).

***Capillaries*** appear behind the sphincters of the arterioles and are the smallest blood vessels (5–10 µM). Due to their thinness, they do not present tunics and are the organ of the circulatory system with the highest level of gas and nutrient exchange. There are three types of capillaries: continuous, fenestrated, and sinusoids according to the specialized subpopulations of EC (114, 116, 126).

***Venules*** are structurally and functionally similar to the capillaries. The postcapillary venules are the main vessel involved in the extravasation of leukocytes to the tissues. Here, endothelial junctions are very labile and sensitive to inflammatory mediators, which makes hyperpermeability processes possible (114, 117, 127).

***Veins*** accumulate a larger volume of blood as they have lower pressure than arteries, and the blood is moved by the skeletal muscular system rather than by the heart (117, 128). They are classified into three types depending on their diameter, which increases as they approach the heart: small (0.1–1 mm), medium (1 mm–1 cm), or large (>1 cm) (116, 129, 130). Therefore, the venous system has additional venous valves formed by folds of the *tunica intima* and whose function is to prevent blood reflux (116, 117, 129, 131).

All blood vessels have a common structure that is mainly composed of three layers, i.e., *tunica intima*, *tunica media*, and *tunica adventitia* (*ti*, *tm*, and *ta*, respectively), but morphological and functional differences exist between them. The *ti* consists of a single and extensive layer of endothelial cells (ECs) that forms a physical barrier between blood and tissues, allowing the selective transport of molecules through it (132). This morphological and functional structure is named the endothelium, and it participates in a multitude of vital functions such as modulating coagulation/fibrinolysis, regulating transportation of inflammatory cells, controlling cellular metabolism, sustaining homeostasis of resident stem cells, guiding organ repair, and releasing inflammatory and angiocrine factors (133, 134). The lumen of the endothelium is covered by a set of proteoglycans and glycoproteins (glycocalyx) which also confer multifunctional and dynamic properties to this layer (135). Thus, *ti* plays a critical role in many physiologic and pathologic processes and as a result the endothelium is considered an organ in itself (136). At the cellular level, ECs are very heterogeneous (137). Their morphology, function, and gene and antigen composition vary between different organs and sections of the vasculature (138, 139). For instance, the capacity of EC to quickly contract in response to inflammatory and vasoactive mediators leads up to endothelial breakdown, resulting in increased vascular permeability, which occurs mainly in the microvasculature (37, 140–143). At the molecular level, two main canonical pathways regulate endothelium stability: the actin-myosin cytoskeleton and those molecular processes related to the tight and adherent junctions (144–147).

The next tunica, *tm*, is located in the middle area of the vessels determining their diameter and is usually composed of several layers of vascular smooth muscle cells (VSMCs) and/or elastin fibers supported mainly by a collagen matrix. VSMCs are fundamental homeostatic players in different diseases such as hypertension and atherosclerosis (148, 149). These cells contain the main molecular machinery to directly regulate the vascular tone (dilatation and constriction) mainly by changes in their intracellular Ca^2+^ (iCa^2+^) levels. Indirectly and with the main purpose of maintaining homeostasis, ECs also contribute to vascular tone modulation *via* synthesis and release of vasoactive substances (150, 151). Specifically, the main relaxant product released by ECs is nitric oxide (NO) (152–154).

The outer layer of the vessel is the *ta*, which is responsible for the integrity and resistance to physical stress of the vascular wall. This layer consists mainly of connective tissue and contains *vasa vasorum.* These small vessels irrigate the whole wall of the large vessel since nutrients and oxygen cannot reach all cells by diffusion (114, 116, 155–157). In addition, this layer might present nerve fibers which regulate vascular tone and which are also altered during pathological processes (158).

## Cardiovascular Pathophysiology of Anaphylaxis

**Figure 3:**
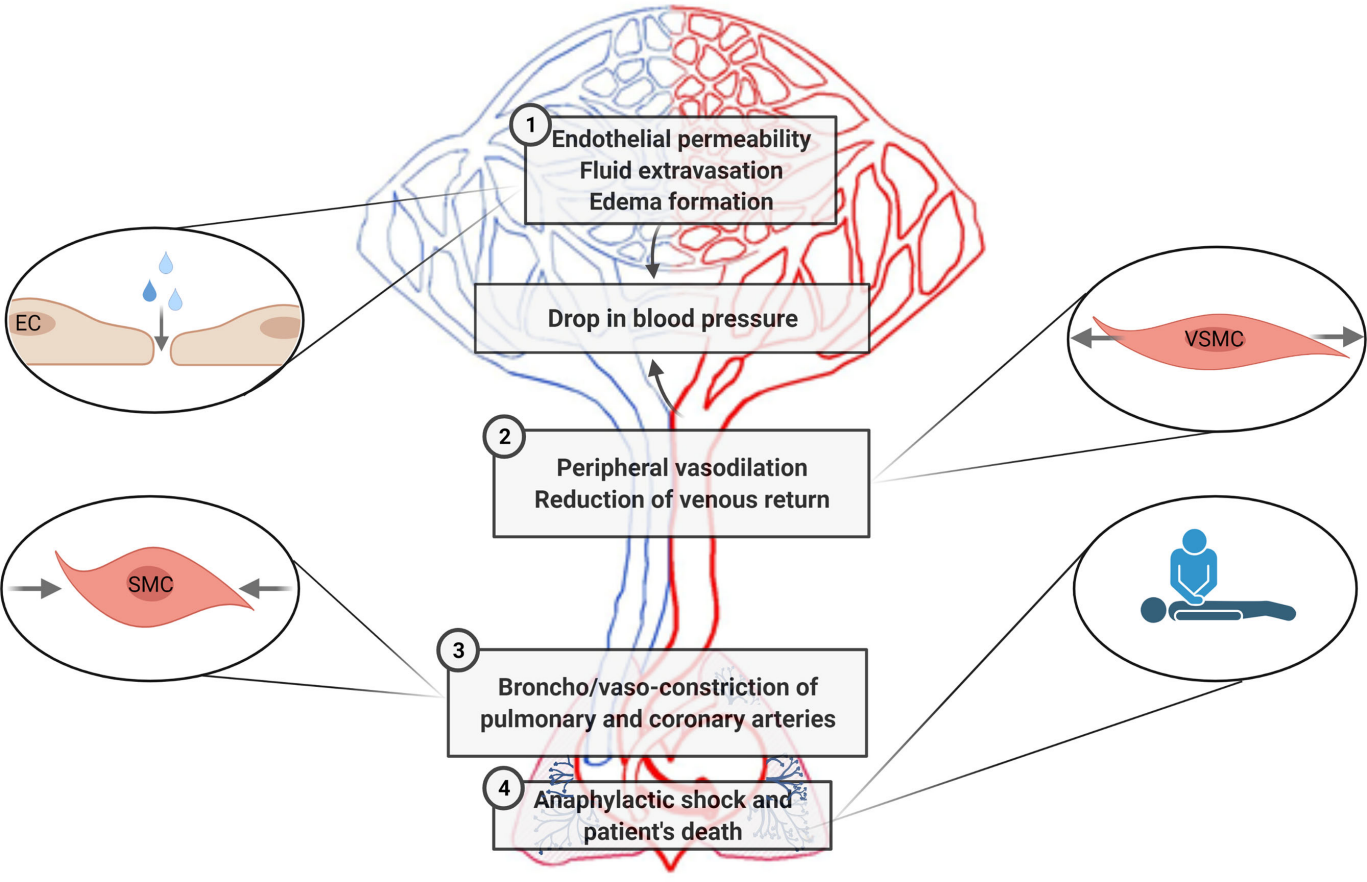
Cardiovascular pathophysiological manifestations in anaphylaxis are sketched according to the main areas/type of vessels/organs corresponding to the processes affected. 1. Increased fluid extravasation (vascular permeability/leakiness)—microcirculation (capillaries and venules). 2. Profound systemic vasodilation (decreased peripheral vascular resistance)—peripheral vessels. 3. Vasoconstriction of the thoracic cavity—coronary and pulmonary arteries. 4. Tachycardia, reduced myocardial contractility and decreased cardiac output—heart. EC, endothelial cells; SMC, smooth muscle cells; VSMCs, vascular smooth muscle cells.

**Figure 4:**
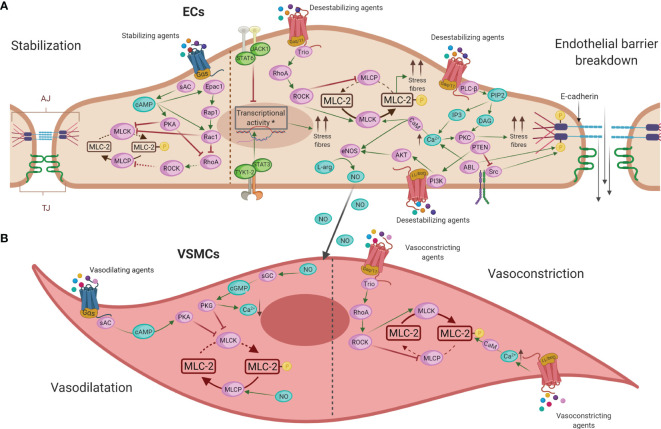
Main intracellular molecular mechanisms in ECs **(A)** and VSMCs **(B)** during anaphylaxis. **(A)** Barrier-stabilizing agents, through adenylate cyclase (AC) and increased levels of cyclic adenosine monophosphate (cAMP), reinforce the actin cytoskeleton in ECs by reducing vascular permeability. On the other hand, the action of anaphylactic mediators through G protein–coupled receptors (GPCRs) induces contraction of ECs, destabilizing the endothelial barrier and increasing vascular permeability by augmenting the cytosolic calcium (Ca^2+^) concentration and the consequent disruption of intercellular junctions in these cells. **(B)** NO induces vasodilation by the activation of guanylate cyclase (GC) in VSMCs, which causes a fall in Ca^2+^ concentration leading to relaxation of these cells. The mechanism underlying vasoconstriction is mediated primarily by increased Ca^2+^, which results in enhanced acto-myosin contractility. EC, endothelial cell; VMSC, vascular smooth muscle cell; TJ, tight junctions; AJ, adherent junctions; AC, adenylate cyclase; cAMP, cyclic adenosine monophosphate; PKA, protein kinase A; Epac1, exchange factor directly activated by cAMP; Rap1, Ras-related protein 1; Rac1, Ras-related C3 botulinum toxin substrate 1; RhoA, Ras homolog family member A; GPCRs, G protein–coupled receptors; Ca^2+^, calcium; PLC-β, phospholipase-C; PIP2, phosphatidylinositol 4;5-bisphosphate; IP3, 1;4;5-trisphosphate; DAG, diacylglycerol; PKC, protein kinase C; CaM, calmodulin; MLCK, MLC kinase; MLC-2, myosin light chain 2; MLCP, myosin light chain phosphatase; ROCK, Rho kinase; JAK, Janus kinase; TYK1-2, non-receptor tyrosine-protein kinase 1 and 2; STAT, signal transducer and activator of transcription; Trio, triple functional domain protein; PTEN, phosphatase and tensin homolog; PI3K, phosphoinositide 3-kinases; Akt, protein kinase B; eNOS, nitric oxide-endothelial synthase; L-arg, L-arginine; NO, nitric oxide; sGC, soluble guanylate cyclase; cGMP, cyclic-guanidine monophosphate; PKG, protein kinase G.

Decades of investigations have demonstrated the importance of specific interactions between immune and vascular cells in different diseases (159). In anaphylaxis, the participation of immune cells leading to the final release of mediators is very well defined (42). Nevertheless, their impact throughout the vascular niche is less clear. Both the rupture of the endothelial barrier and vascular tone disturbances are the essential anomalies observed during this pathologic event. In addition, phenomena occurring in the chest cavity (e.g., cardiac arrest, decreased cardiac output, hypoxia) are present in anaphylactic reactions (**Figure 3**). Molecularly, the EC- and VSMC-signaling pathways govern these processes (**Figure 4**). A dysfunctional endothelium is the cause of important cardiovascular diseases such as thrombosis, atherosclerosis, or hypertension. Even a damaged endothelium has been observed in patients with mastocytosis (160). Additionally, in acute inflammatory situations, as it has been observed in COVID-19 disease, ECs contribute as effector cells to the cytokine storm (161). In addition, the endothelium actively participates in the activation of the coagulation, contact, and complement in anaphylaxis (44, 162, 163). Therefore, its study is a hot topic and of great importance for the treatment of several pathologies such as anaphylaxis.

### Increased Vascular Permeability and Endothelial Barrier Breakdown

For years, anaphylaxis has been associated with a concomitant leakage of fluids (164). A highly relevant investigation employing indirect measurements of changes in hemoglobin concentration demonstrated that a transfer of up to 35% of the fluid to the extracellular space can occur within 10 min of allergen exposure (165). Another interesting study carried out in mice determined that histamine-induced vascular permeability associated with anaphylaxis occurs in postcapillary venules (166). Furthermore, a recent investigation focusing on humans has shown the relevance of micro-ECs as the sole responders to anaphylactic mediators in *in vitro* vascular permeability (167). The high extravasation of fluid contributes to edema of the airways and pulmonary emphysema (168). Specifically, a study including 50 idiopathic anaphylaxis subjects showed that upper airway obstruction was strongly associated with laryngeal edema, possibly leading to asphyxia (169). In addition, investigations based on systemic anaphylaxis using allergic rats observed an association between interstitial airway vascular leakage and severe bronchoconstriction (170). On the other hand, angioedema and urticaria, the most common symptoms of anaphylaxis, involve vascular fluid extravasation and are characterized by temporary localized swelling (171, 172). Angioedema can affect all layers of the skin and visceral walls, such as the respiratory system and the gastrointestinal tract (171, 173, 174). Furthermore, angioedema of the upper airways or pulmonary edema has been observed in one out of two human anaphylactic biopsies indicating the necessity to control vascular permeability (18). Importantly, the endothelial IL-4 receptor α chain (IL-4Rα) and its underlying signaling appears as relevant in the gastrointestinal extravasation disturbances associated with food allergic reactions (175). A recent study carried out in peanut-challenged anaphylactic patients has shown that most leakage volume takes place in the gut, contributing to the appearance of gastrointestinal symptoms (176). Furthermore, intestinal extravasation has also been determined in a drug-induced anaphylactic patient in whom a diffuse edema was observed (177).

Molecularly, stabilization of the endothelial barrier depends on a series of connections, such as tight junctions (TJ) and adherent junctions (AJ). TJs are mainly composed of occludins and claudins that bind to the actin cytoskeleton and α-catenin. In AJs, VE-cadherin is the major structural protein, and they contribute to barrier stabilization by providing mechanical cohesive strength between ECs (178, 179). Different known anaphylactic mediators such as histamine and PAF are widely described as inductors of vascular permeability (46). Their action through its endothelial receptors activates phospholipase C (PLC-β) which hydrolyzes phosphatidylinositol 4,5-bisphosphate (PIP2) to produce two second messengers: inositol 1,4,5-trisphosphate (IP3) and diacylglycerol (DAG). IP3 raises intracellular iCa^2+^ levels stimulating calcium-regulated mechanisms. This iCa^2+^ acts, together with DAG, to activate protein kinase C (PKC) that contributes to the disruption of junctional protein complexes. In addition, iCa^2+^/calmodulin (CaM) activates myosin light chain kinase (MLCK) leading to the phosphorylation of myosin light chain 2 (MLC-2) and resulting in increased acto-myosin contractility, stress fibers, and the disruption of the endothelial barrier. In turn, myosin light chain phosphatase (MLCP) dephosphorylates MLC-2 producing cellular stability. MLCP inhibition is achieved by Rho kinase (ROCK) activation downstream of the guanine nucleotide exchange factor, Trio, which initiates the activation of RhoA (46, 178, 180, 181). In addition, the non-receptor tyrosine kinases, ABL and Src, are activated in response to anaphylactic stimuli contributing also to iCa^2+^ mobilization and VE-cadherin dissociation (182, 183). Moreover, other novel endothelial molecules also participate in leakage processes. The fibroblast growth factor-inducible 14 (Fn14) receptor, the signal transducer and activator of transcription 3 (STAT3), the peroxisome proliferator-activated receptor (PPAR β/δ), and MALT1 protease activity have been proposed as therapeutic strategies in allergy and anaphylaxis (84, 175, 183–185). In addition, recently, a challenge study has demonstrated the capacity of EVs purified from plasma of anaphylactic patients to interact with ECs, eliciting vascular permeability and thereby suggesting that cellular communication between microenvironments may also be established in anaphylaxis (186).

In contrast, other mediators stabilize the endothelial barrier, mostly to maintain homeostasis. These barrier-stabilizing agents, through adenylate cyclase (AC), increase cyclic adenosine monophosphate (cAMP) levels in ECs. It activates the protein kinase A (PKA) and guanine nucleotide exchange factors such as Rac1 which lead to inhibition of the small GTPase RhoA and the strengthening of the actin cytoskeleton stabilizing the endothelial barrier (46, 178). Specifically, sphingosine-1-phosphate (S1P) and prostaglandin D2 (PGD2) are some of these soluble stabilizing molecules while intracellularly phosphatidylinositol 3-kinase (PI3K-C2α) and the regulator of calcineurin 1 (Rcan1) have been argued to strengthen the endothelium (187–193).

### Vasodilation and Hypotension

Adequate control of peripheral vascular pressure is essential, as it provides the driving force to pump blood to the organs (194). Vasodilation is predominantly controlled by the autonomic nervous response (195). However, anaphylactic mediators can directly contribute to the loss of the vascular resistance even in the absence of neural input. Vasodilation has been observed in anaphylactic patients with severe cardiovascular manifestations such as hypotension (16, 165, 196). The drop of blood pressure has been attributed both to fluid extravasation and to loss of vascular resistance, rather than a direct effect of the myocardium (165). Specifically, a study points to the action of mediators leading to vasodilation and increased vascular permeability (197). Therefore, it remains unclear whether vasodilation is derived from or leads to fluid leakage in human anaphylaxis. To shed light on this, an interesting study using an experimental murine model found a correlation between the increase in vascular permeability and a fall in blood pressure during early anaphylaxis. This fact suggests that fluid leakage may act as a precursor of hypotension and have shared molecular mechanisms (198). On the other hand, vasodilation causes excessive venous blood accumulation due to the significant reduction in venous return that occurs during anaphylaxis (199). It is consequent to think that veins, which are the reservoirs of blood in the body, undergo serious functional impairment during this event. Specifically, hemodynamic monitoring in a clinical case of anaphylactic reaction to penicillin indicated a reduction in cardiac output owing to this decrease in venous return (200). Therefore, the impact of the altered flow leads to cardiac compromise and suggests that veins are an important niche to study (199, 201).

In anaphylaxis, the main anaphylactic mediators not only play a role in vascular permeability but also regulate the tone of the vessel. At the cellular and molecular levels, the activation of the endothelial nitric synthase (eNOS) and the underlying production of NO have been probably characterized as the most harmful vasodilator and hypotensive factor (187, 202–204). This molecule induces vasodilation by the activation of soluble guanylate cyclase (sGC) in VSMCs, causing increased formation of cyclic-guanidine monophosphate (cGMP). Subsequently, it activates protein kinase G (PKG), which causes a fall in iCa^2+^ concentration, ensuring that MLCK can no longer phosphorylate MLC-2 and leading to relaxation of the VSMCs and vasodilation. Furthermore, NO activates MLCP and, by dephosphorylating MLC-2, causes additional relaxing effects (46, 204). Anaphylactic patients with respiratory manifestations associate with increased levels of exhaled NO (205). In addition, this mediator could act as a regulator of microvascular permeability (152–154). Therefore, the endothelium participates in modulating the resistance of the vessels and VSMCs contribute to maintaining a correct vascular tone and the consequent homeostasis. Furthermore, other mediators with vasodilator capacity have been described in this context. In the case of histamine, its contractile and dilating effects depend on the expression and activity of the histamine receptors located on both ECs and VSMCs (206, 207). Tryptase modulates vasodilation through calcitonin genes and by the release of neuromodulators such as substance P (208–210). Bradykinin, a hypotensive factor released during anaphylaxis as a result of contact system activation, leads to vasodilation and associates with laryngeal edema (109).

### Vasoconstriction

The regulation of the vascular system presents a dilemma because a third block of important circulatory disturbances may also appear in anaphylaxis. Severe reactions seem to present vasoconstriction of the vessels in the thoracic cavity leading to cardiac arrest (211). The coronary and pulmonary arteries are the primary vessels susceptible to contraction (150, 212). However, constrictive processes carried out by the main underlying anaphylactic mediators are neither clearly described nor studied in human anaphylaxis (213–216), except increased serum troponin that is observed in Kounis syndrome and Takotsubo cardiomyopathy (211). Therefore, due to the obvious complications of studying these processes in emergency situations, most of the available evidence is based on animal studies. Vasoconstriction of the pulmonary artery has been associated with right ventricular failure in animal models, favoring the circulatory collapse seen in anaphylaxis (217). On the other hand, left ventricular dysfunction has been related to coronary vasoconstriction in a rat model (218). Additionally, another rat model of anaphylaxis revealed increased portal venous resistance and, therefore, hepatic vasoconstriction (219). There is also a contraction of the bronchial tree that hinders gas exchange in the alveoli, generating hypoxia. This fact, in turn, supports the constriction of the pulmonary circulation compromising the entire vascular system and has been associated with bronchospasm and death in guinea pigs (220, 221). Therefore, in anaphylaxis, vasoconstriction of pulmonary and coronary arteries contributes to the reduction of myocardial contractility and may be a major cause of death (196, 211, 212).

Underlying mechanisms to vasoconstriction are mostly mediated by increased iCa^2+^ which activates MLCK leading to the phosphorylation of MLC-2 and resulting in increased acto-myosin contractility (148).

### Anaphylactic Shock

The previous described pathophysiological manifestations (increased endothelial permeability, peripheral vasodilation, and thoracic cavity constriction) alter blood flow, resulting in impaired vascular homeostasis, which may lead to anaphylactic shock (171, 197).

The exacerbated fluid extravasation, in combination with the loss of peripheral vascular resistance, reduces venous return to the heart. In addition, the blood flow is removed from surface areas, reducing oxygen demands and delivering it to vital organs, thereby decreasing body temperature. However, it is insufficient to satisfy the metabolic demands of the human organism. On the other hand, constriction of pulmonary arteries, together with the effect of tightened airway smooth muscle, leads to reduced oxygen uptake. Therefore, to make up for this lack of oxygen in the body, breathing becomes rapid and deep and the heart rate increases (tachycardia). However, a second phase characterized by bradycardia occurs because this organ cannot remedy the influence of the previous manifestations of this pathological event, causing cardiac collapse and death of the patient (171, 201, 222, 223).

## Membrane Receptors in Anaphylaxis

A wide variety of soluble mediators have been proposed as participants of the anaphylaxis pathophysiology (46, 71). The majority of these molecules are ligands for the largest group of membrane receptors which are those linked to heterotrimeric G proteins (GPCRs). Additionally, they have been implicated in a variety of diseases (224–226). However, GPCR downstream signaling pathways are not well characterized for every anaphylactic mediator, although their impact on vascular permeability and vasodilation is well established (**Table 2**).

**Table 2:** Main vascular GPCRs in anaphylaxis.

Mediators	Receptors	Vascular permeability	Vasodilation/ Hypotension	Vasoconstriction	References
**Histamine**	HR1 (Gα_q/11_)	X	X	X	(181, 206, 207, 227–229)
	HR2 (Gα_s_)	X	X	X	
**PAF**	PAF-R (Gα_q/11_)	X	X	X	(24, 230, 231)
**Tryptase**	PAR2 (Gα_q/11_)	X	X	X	(208, 232, 233)
**LTB4, LTC4, LTD4, LTE4**	CysLT1R (Gα_q/11_) CysLT2R (Gα_q/11_)	X	X	X	(234–236)
		X	X	X
**PGD2**	DP (Gα_s_)	X	X		(71, 192)
**PGE2**	EP1 (Gα_q/11_)		X	X	(237)
	EP2 (Gα_s_)		X	X
	EP4 (Gα_s_)			X	X
**PGF2**	FP (Gα_q/11_)			X	(238)
**TXA2**	TP (Gα_q/11_)			X	(238)
**Bradykinin**	BR1 (Gα_q/11_)	X	X	X	(109, 163, 239)
	BR2 (Gα_i_)	X	X	X	
**S1P**	S1PR1 (Gα_i_)		X	X	(187, 189, 190, 240, 241
	S1PR2 (Gα_i/o,_ Gα_q/11,_ Gα_12/13_)		X	X		
	S1PR3 (Gα_i/o,_ Gα_q/11,_ Gα_12/13_)		X	X	
**C3a**	C3aR (Gα_q/11_)	X	X	X	(242–244)
**C5a**	C5aR (Gα_i_)	X	X	X	
**Epinephrine**	α1AR (Gα_q/11_)		X	X	(245, 246)
	α2AR (Gα_i_)		X	X		
	β2AR (Gα_s_)		X	X		

The processes affected by those most described anaphylactic mediators are listed. They signal through different G protein-coupled receptors (GPCRs) which address their intracellular signaling through different pathways that ultimately lead to the main cardiovascular alterations in anaphylaxis (vascular permeability, vasodilation/hypotension, or constrictive processes).

In this sense, most of the studies have mainly been carried out in experimental models (37). Specifically, anaphylactic shock depending on endothelial Gq/G11 was characterized in mice (247).

## Treatment

**Figure 5:**
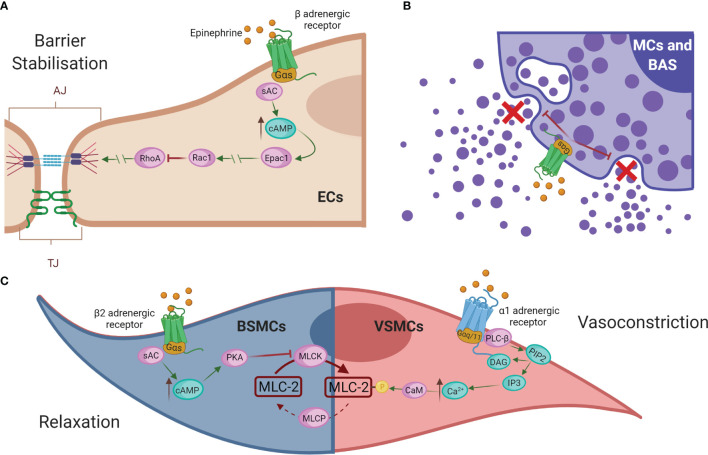
Epinephrine regulates vascular permeability and resistance. **(A)** Epinephrine promotes EC barrier stability through β-adrenergic receptors. **(B)** In addition, β2-adrenergic receptors regulate the inhibition of degranulation of mediators released by the effector cells of the immune system, mast cells (MCs), and basophils (BAS). **(C)** In bronchial smooth muscle cells (BMSCs), epinephrine restores bronchoconstriction by relaxing the contraction signal, whereas in VSMCs it exerts a vasoconstrictor action *via* α-adrenergic receptors. AC, adenylate cyclase; cAMP, cyclic adenosine monophosphate; Epac1, exchange factor directly activated by cAMP; Rac1, Ras-related C3 botulinum toxin substrate 1; RhoA, Ras homolog family member A; EC, endothelial cell; MCs, mast cells; BAS, basophils; BSMC, bronchial smooth muscle cells; PKA, protein kinase A; MLCK, MLC kinase; MLC-2, myosin light chain 2; MLCP, myosin light chain phosphatase; VMSC, vascular smooth muscle cell; PLC-β, phospholipase-C; PIP2, phosphatidylinositol 4;5-bisphosphate; DAG, diacylglycerol; IP3, 1;4;5-trisphosphate; Ca^2+^, calcium; CaM, calmodulin.

To date, the first-line and most effective anaphylaxis treatment is intramuscular administration of adrenaline/epinephrine (245). It is indicated after recognition of symptoms due to its ability to directly remedy alterations in the cardiovascular system caused by the reaction. Its administration prevents cardiovascular collapse and enhances blood flow during anaphylactic shock (12, 248, 249). Mechanistically, this drug resolves anaphylaxis *via* its different adrenergic receptors (**Figure 5**). Epinephrine exerts its vasoconstrictor action through α-adrenergic receptors on VSMCs. Its activation causes stimulation of PLC-β, which cleaves PIP2 into DAG and IP3. This increased intracellular IP3 binds to its receptors, leading to higher iCa^2+^ levels and subsequent myosin phosphorylation (250, 251). The result of this pathway is a peripheral vasoconstriction that reverses peripheral vasodilation alleviating hypotension and reducing edema (252, 253). On the other hand, epinephrine, through β-adrenergic receptors and subsequent Gα(s) activation, results in enhanced AC activity, which promotes cAMP formation and the stability of the ECs barrier (254). In addition, through β1 receptors it increases heart rate and its force of contraction, while β2 receptor-mediated action restores bronchoconstriction and reduces the release of inflammatory mediators by the main immune effector cells, mast cells, and basophils (251, 252, 255). In bronchial smooth muscle cells (BSMCs), epinephrine restores its constriction through the β2-adrenergic receptor. cAMP production activates PKA, which phosphorylates and inactivates the MLCK. These facts stop the downstream signal for contraction and thus relax BSMCs (251). However, this drug administration is not always effective and patients may still die.

Epinephrine would be ineffective in cardiopathy patients receiving β-blockers, and glucagon is occasionally administered. The action of this drug is not mediated by adrenergic receptors and allows reversal of refractory hypotension and bronchospasm (1, 249). Moreover, there are other complementary therapies which may be useful but should never replace the first-line treatment. These include supplemental oxygen, β2-agonists to reverse bronchospasm, intravenous crystalloid solutions to restore circulatory volume, antihistamines to control cutaneous symptoms, and glucocorticosteroids to prevent biphasic reactions and reduce inflammation (256).

Currently, various investigations on potential alternative drugs for the treatment of anaphylaxis are being conducted, although they are not implemented in clinical routine yet. Methylene blue is an inhibitor of NO pathways and has been reported to be a safe medication for refractory hypotension underlying anaphylactic shock (257). On the other hand, sugammadex, a g-cyclodextrin, has been shown to encapsulate and inactivate neuromuscular blocking agents, which may be triggers of this pathological event. However, its usefulness remains controversial since a recent study of perioperative anaphylaxis treated with this drug concluded that it did not modify the reaction (258). In addition, promising results have been obtained from mouse models where PAF antagonists have been shown to mitigate the severity and duration of anaphylaxis (259).

Otherwise, desensitization interventions have been proposed for the long-term treatment and prevention of anaphylaxis (260). Specifically, biological agents are emerging as a potential adjuvant for this process (36, 261). Among them, omalizumab, a monoclonal antibody against IgE, has been shown to be a successful treatment in reducing the number and severity of anaphylactic reactions (262). In addition, several therapies are appearing for the prevention of food-induced anaphylaxis. These are aimed to modulate specific immune pathways in different ways, such as antibodies against the main cytokines involved in the response (anti-IL-33, anti-IL-5, etc.) or immunotherapies to polarize the Th2 response characteristic of allergic reactions to Th1 and T regulatory (Treg) ones through toll-like receptor (TLR) agonists (TLR4 and TLR9), nanoparticles, probiotics, or IFNγ, among others (263–265).

## Conclusions

Understanding the extent of human vessels in anaphylaxis is the challenge we have taken on in this study. Although the vascular system functions as a whole, specific features are identified in the different types of vessels. Their specialization is based on the morphology, functionality, and molecular signaling pathways of the vascular cells composing those regions (138). Therefore, expanding the knowledge of human vessels in anaphylaxis is crucial to implementing tools based on their underlying molecular mechanisms. It would help to achieve better quality of acute and long-term clinical management of anaphylactic patients.

For years, the classic cellular and molecular mechanism described for anaphylaxis has been the cross-linking of FcϵRI-bound allergen–IgE complexes activating mast cells/basophils and the consequent release of mediators (5). More recent studies shed light about the relevance of other immune effector cells in anaphylaxis such as monocytes, macrophages, neutrophils, eosinophils, and platelets (266). Unfortunately, most of this evidence is based on murine models but knowledge about its involvement in human anaphylaxis still remains scarce (12). Due to the features of these innate immune cells, the main research focus in molecular anaphylaxis is on the immune system. However, anaphylaxis is a systemic reaction in which several organs and systems are involved. Therefore, simultaneously to the inflammatory response, the vasculature actively participates in such pathophysiological process. Epinephrine is the treatment of choice to palliate anaphylactic symptoms accordingly to guidelines (245). It is the “safeguard” molecule which triggers both endogenous and exogenous mechanisms through its adrenergic receptors (1). Therefore, the downstream signaling pathways of these receptors involve a dual behavior (constriction and relaxation processes) in vascular and bronchial smooth muscle cells. In addition, both cell types, either located within the vessel wall or conforming to organs, would relate to the variety of clinical consequences and the severity grade of the reactions.

In this context, the wide endothelium surface would also be contributing differentially to the pathophysiology of the anaphylaxis. The endothelium arises as an important *organ-cell like* in anaphylaxis playing a role not only in the control of fluids and the vascular tone but also as an activation surface for the coagulation, contact, and complement systems (44, 162, 163). Moreover, ECs release relevant anaphylactic mediators such as NO, although it is likely that other molecules (presumably cytokines or interleukins) could also be released contributing to the pool of mediators in the reaction. However, the exact contribution of these cells as provider of mediators to the anaphylactic cellular microenvironments existing between immune and resident niches is unknown.

Altogether, we can summarize that the permeable and/or vasomotor capacity of the different anaphylactic mediators (NO, tryptase, histamine, bradykinin, or a multitude of other scarce or unknown ones) would determine the magnitude of the anaphylactic events. Furthermore, the role of their receptors (GPCRs) in human anaphylaxis is not yet fully understood, but they result to be essential in preventing or inducing vascular effects. Therefore, GPCRs are the most promising therapeutic targets of study nowadays and the degree of responsiveness of ECs and VSMCs is an important factor to determine in anaphylaxis.

Due to the complexity to investigate in human subjects the cellular and molecular mechanisms of anaphylaxis, the improvement and reproducibility of animal—or *in vitro* human—models is necessary. This fact is one of the major limitations to study this pathological event. A big piece of the literature around it derives from animal models. For that reason, conclusions must be taken cautelous when translated at human reactions. Strategies based on functional *in vitro* studies by using human cell cultures combined with sera would provide clues about cellular and molecular happenings occurring in specific anaphylactic microenvironments. Therefore, abundant investigations are aimed at finding vascular targets revealing future alternative strategies to treat or prevent anaphylaxis. Efforts must be focused on alternative therapies called to specifically modulate the whole or parts of the vascular system that benefit the clinical management of these life-threatening events in the near future.

## Author Contributions

Conceptualization and—original draft preparation, VE. Writing—review and editing, EN-B, SF-B, AY-M, and VE. Funding acquisition, VE. All authors contributed to the article and approved the submitted version.

## Funding

This research was supported by grants from the Instituto de Salud Carlos III (PI18/00348 and PI21/00158) and FEDER Thematic Networks and Cooperative Research Centers RETICS ARADyAL RD16/0006/0013. This work was also supported by the SEAIC (19_A08) and Alfonso X el Sabio University Foundations. EN-B was granted by funding from the Community of Madrid included in the project FOOD-AL (CM_P2018/BAAA-4574).

## Conflict of Interest

The authors declare that the research was conducted in the absence of any commercial or financial relationships that could be construed as a potential conflict of interest.

## Publisher’s Note

All claims expressed in this article are solely those of the authors and do not necessarily represent those of their affiliated organizations, or those of the publisher, the editors and the reviewers. Any product that may be evaluated in this article, or claim that may be made by its manufacturer, is not guaranteed or endorsed by the publisher.
